# Some further results on the stability of Ky Fan’s points

**DOI:** 10.1186/s13660-017-1562-1

**Published:** 2017-11-21

**Authors:** Shuwen Xiang, Jihao He, Chengwei Liu, Wensheng Jia, Yanlong Yang

**Affiliations:** 1 College of Computer Science and Technology, GuiZhou University, Guiyang, Guizhou 550025 China; 2 School of Mathematics and Statistics, GuiZhou University, Guiyang, Guizhou 550025 China

**Keywords:** 26B25, 47H10, 54A05, 54H25, 91A10, 91A40, 91A44, Ky Fan’s points, section mappings, maximum Hausdorff semi-metric, essential component, Nash equilibrium

## Abstract

In this paper, some further results on the stability of Ky Fan’s points are proposed by introducing a type of stronger perturbation of section mappings defined by a semi-metric called the maximum Hausdorff semi-metric, and the existence of the essential components of the set of Ky Fan’s points to this perturbation is proved. As an application, the existence of the essential component of the Nash equilibrium is presented using the proposed method, and strong robustness to payoff function perturbation is achieved.

## Introduction

Let us consider a function $f:X\times X\rightarrow R $, where *X* is a nonempty compact convex subset of a Hausdorff topological linear space.

The Ky Fan minimax inequality (Ky Fan’s inequality, for short, [1]) problem, denoted by (**KF**), consists in finding an element $y^{\ast}\in X$ such that

$$f\bigl(x,y^{\ast}\bigr)\leq0 \quad(x\in X). $$

The function *f* is called an inequality function, and the element $y^{\ast}$ is called a Ky Fan point.

It is well known that Ky Fan’s inequality theorem plays a very important role in the research field of nonlinear and convex analysis [2]. Because of the wide application of Ky Fan’s inequality in optimization, convex analysis, variational inequality, control theory, fixed point theory and mathematical economics, it has been generalized in various ways, such as the implicit variational inequality, equilibrium problem, vector variational inequality and mixed implicit variational inequality ([3–14]). Because Ky Fan’s inequality is closed related to optimization, variational inequalities, the fixed point problem and many other problems after 2015, the corresponding algorithms have been studied in [15–17].

Because Ky Fan’s point is equivalent to the Nash equilibrium, it has become a powerful tool to study the noncooperative game problem. The Nash equilibrium is a core concept of noncooperative games, and it has extensive applicability, which extends well beyond economics and other behavioral sciences. However, it has become clear that the concept of equilibrium has some serious drawbacks that limit its usefulness. First, there is often more than one equilibrium, and, in some cases, there is a very large (or even infinite) number. The problem of multiple equilibria makes it unclear which equilibrium players should focus on, because there is no reasonable way to decide which equilibrium will be selected. In addressing the multiplicity of Nash equilibria, game theorists have examined a variety of arguments that refine the set of equilibria ([13, 18–20]). The refinement of equilibria should follow a certain rational principle; thus, a reasonable approach for refinement, as used in many concepts, is to select an equilibrium that is ‘stable’ to a slight perturbation caused by uncertainty in the game. Therefore, stability has become an important method for refining the Nash equilibrium ([14, 21–26]). Kohlberg and Mertens [22] proposed the concepts of the KM equilibrium based on various ‘stable’ sets of the Nash equilibrium.

The stability of the Nash equilibrium mainly focuses on the ‘stable’ set (or point) with respect to the perturbation of the strategy sets or payoff functions. However, it is difficult to directly study the stability of the Nash equilibrium from the game models themselves. A current method is to study the stability of the Nash equilibrium by means of the equivalence between the Nash equilibrium and solution of some nonlinear problems, for example, it is studied by applying the fixed point of the best reply correspondence. The defect that exists in the method of the best reply correspondence is the discontinuity between the best reply correspondence and the strategy set or payoff function; that is, in such results, it cannot be stated whether the Nash equilibrium is ‘stable’ with respect to the perturbation of the strategy sets or payoff functions.

Another approach that considers the stability of the Nash equilibrium is based on Ky Fan’s point. Studies on the stability of Ky Fan’s point have been conducted. Recently, Tan *et al.* [23] proposed the generic stability of Ky Fan’s point with respect to the perturbation of inequality functions equipped with the sup-norm metric. Yu *et al.* [24] and [25] proved the existence of essential components with respect to the same perturbation form. Additionally, as is well known, Ky Fan’s section theorem is equivalent to Ky Fan’s inequality, but there is no longer any function in its form of expression. To study the stability of Ky Fan’s section theorem, Zhou *et al.* [26] introduced a maximum Hausdorff metric of section mappings and proved the existence of the essential component of the set of solutions of Ky Fan’s section theorem, which was another approach to set up an alternative stable set of Ky Fan’s point defined by the perturbation of section mappings. In both these cases, two types of perturbation were defined by the sup-norm of the inequality functions and the maximum Hausdorff metric of section mappings, respectively. Despite this, an example (Example 4.1, [26]) shows that there is no direct relationship between these two types of perturbation.

Therefore, there is still an interesting question that deserves attention: Is it possible to establish a type of stronger stability in which a stronger ‘stable’ set is stable with respect to the two aforementioned perturbations? To address this question, in this paper, the stability of Ky Fan’s points is established by introducing a type of stronger perturbation of section mappings defined by a semi-metric called the maximum Hausdorff semi-metric, which includes the two aforementioned perturbations. In this paper, the existence of the essential component of the set of Ky Fan’s points with respect to the stronger perturbation is proved. An example of the Nash equilibrium is given to illustrate our results.

## Preliminaries

Let *X* be a nonempty convex compact set in a normed linear space, and *d* be a metric defined on $X\times X$, then $d(x,y)$, denoted by $(\| x_{2}-x_{1}\|+\| y_{2}-y_{1}\|)$, is the distance between two points $(x_{1},y_{1})$, $(x_{2},y_{2})$ in $X\times X$, and $H_{d}$ is the Hausdorff metric defined on *X* or $X\times X$.

Firstly, we present the classical Ky Fan’s section theorem.

### Theorem A

([2])

*Let* *X* *be a nonempty compact convex set in a normed linear space*. *Let A be a subset of* $X\times X$ *with the following properties*:

1. *For any fixed* $x\in X$, *the set* $\{y\in X:(x,y)\in A\}$ *is closed*.
2. *For any fixed* $y\in X$, *the set* $\{x\in X:(x,y)\notin A\}$ *is convex* (*or empty*).
3. $(x,x)\in A$ *for every* $x\in X$.

*Then there exists a point* $y_{0}\in X$ *such that* $X\times\{y_{0}\} \subset A$. The point $y_{0}$ is called solution of Ky Fan’s section theorem with respect to *A*.

We introduce now an alternative form of Ky Fan’s section theorem, *i.e.*, the famous Ky Fan’s inequality theorem.

### Theorem B

([1])

*Let* *X* *be a nonempty compact convex set in a normed linear space*. *Let* $\varphi: X \times X \rightarrow R$ *be a function with the following properties*:

1. *For any fixed* $x\in X$, $y\rightarrow\varphi(x,y)$ *is lower semicontinuous*.
2. *For any fixed* $y\in X$, $x\rightarrow\varphi(x,y)$ *is quasiconcave*.
3. $\varphi(x,x)\le0$ *for every* $x\in X$.

*Then there exists a point* $y^{\ast} \in X$ *such that* $\varphi (x,y^{\ast})\le0$ ($x\in X$). The point $y^{\ast}$ is called Ky Fan’s point of *φ*.

Denote all nonempty compact and compact convex subsets of *X* by $K(x)$ and $\mathit{CK}(x)$, respectively. Additionally, define

$$\mathcal{A} =\bigl\{ A| A \text{ satisfies conditions (1)-(3) from Theorem A} \bigr\} . $$

Every $A\in\mathcal{A}$ corresponds to a section mapping $E_{A}:X \rightarrow2^{X} $:

$$E_{A}(x)=\bigl\{ y\in X|(x,y)\in A\bigr\} \quad(x\in X). $$

Similarly, define

$$\mathcal{E} =\bigl\{ E_{A}|E_{A}:X\rightarrow K(X),A\in \mathcal{A}\bigr\} . $$

Conversely, every section mapping $E_{A}: X\rightarrow2^{X}$ corresponds to a set

$$A_{E}=\bigl\{ (x,y)\in X\times X|x\in X,y\in E_{A}(x)\bigr\} , $$

and it is easy to verify that $A_{E}\in\mathcal{A}\subset X\times X$.

Further, $y_{0}\in\bigcap_{x\in X}\{y\in X|(x,y)\in A\}=\bigcap_{x\in X}E_{A}(x)$ is solution of Ky Fan’s section theorem with respect to *A*. Denote the set of all solutions of Ky Fan’s section theorem by $F_{s}(E_{A})=\bigcap_{x\in X}\{y\in X|(x,y)\in A\}=\bigcap_{x\in X}E_{A}(x)$. By Theorem A, $F_{s}(E_{A})$ is a nonempty and closed set. Hence, $F_{s}$ is a nonempty compact-valued mapping, *i.e.*, $F_{s}:\mathcal{A}\rightarrow K(X)$.

For Ky Fan’s inequality theorem, define

$$\mathcal{M}=\Bigl\{ \varphi\big| \varphi\text{ satisfies conditions (1)-(3) from Theorem B and }\sup_{(x,y)\in X\times X}\big| \varphi(x,y) \big| < + \infty\Bigr\} . $$

For every $\varphi\in\mathcal{M}$, let $F_{k}(\varphi)=\{y\in X| \varphi(x,y)\le0, x\in X \}$ be the set of all Ky Fan’s points of *φ*. By Theorem B, $F_{k}(\varphi)$ is a nonempty and closed set. Hence, $F_{k}$ is a nonempty compact-valued mapping, *i.e.*, $F_{k}:\mathcal {M}\rightarrow K(X)$.

For every $\varphi\in\mathcal{M}$, define

$$A_{\varphi}=\bigl\{ (x,y)\in X\times X|\varphi(x,y)\leq0\bigr\} , $$

and

$$E_{\varphi}(x)=\bigl\{ y\in X|(x,y)\in A_{\varphi} \bigr\} =\bigl\{ y\in X|\varphi (x,y)\leq0\bigr\} \quad( x\in X). $$

Obviously, $A_{\varphi} \in\mathcal{A}$, $E_{\varphi}\in\mathcal{E} $ and it is easy to verify that $x^{\ast} \in F_{k}(\varphi)$ iff $x^{\ast}\in F_{s}(E_{\varphi})$.

For the study of the stability of Ky Fan’s points, the perturbation is usually defined by the sup-norm metric of the inequality functions naturally. Tan *et al.* [23] proposed the generic stability of Ky Fan’s points, Yu *et al.* [24] and [25] proved the existence of the essential component using this metric. Define the sup-norm metric as follows:

$$\rho_{m}(\varphi_{1},\varphi_{2})=\sup _{(x,y)\in X\times X} \big| \varphi _{1}(x,y)-\varphi_{2}(x,y) \big| \quad( \varphi_{1},\varphi_{2}\in\mathcal{M}). $$

We do not have an inequality function, but a section mapping. In order to study the stability of Ky Fan’s section theorem, Zhou-Xiang [26] defined the maximum Hausdorff metric of the section mappings as $\rho _{s}(E_{A},E_{B})=\sup_{x\in X}H_{d}(E_{A}(x),E_{B}(x))$ ($E_{A},E_{B}\in\mathcal{E}$).

In order to formulate the stability of Ky Fan’s points as an equivalent stability of Ky Fan’s section theorem, the maximum Hausdorff metric of the inequality functions is also defined as $\rho_{1}(\varphi _{1},\varphi_{2})=\rho_{s}(E_{\varphi_{1}},E_{\varphi_{2}})=\sup_{x\in X}H_{d}(E_{\varphi_{1}}(x),E_{\varphi_{2}}(x))$ ($\varphi _{1},\varphi_{2}\in\mathcal{M}$).

Obviously, $(\mathcal{E} ,\rho_{s})$ and $(\mathcal{M},\rho_{1})$ are both metric spaces.

Some fundamental terminologies are presented as follows.

### Definition 1

Let $E\in\mathcal{E} $. A nonempty closed subset $e(E)$ of $F_{s}(E)$ is said to be an essential set of $F_{s}(E)$ with respect to $\rho_{s}$ if, given any number $\epsilon >0$, there exists a corresponding number $\delta>0$ such that $F_{s}(E^{\prime})\cap[e(E)+B_{\epsilon}(0)]\neq\emptyset$ for all $E^{\prime}\in\mathcal{E} $ such that $\rho_{s}(E^{\prime},E)<\delta$.

### Remark 1

(1) If $e(E)=\{y_{0}\}$ is a singleton set and an essential set with respect to $\rho_{s}$, then the point $y_{0}$ is said to be an essential point with respect to $\rho_{s}$.

(2) A connected component $C(E)$ of $F_{s}(E)$ is said to be an essential component of $F_{s}(E)$ with respect to $\rho_{s}$, if $C(E)$ is essential with respect to $\rho_{s}$.

### Definition 2

Let $\varphi\in\mathcal{M}$. A nonempty closed subset $e(\varphi)$ of $F_{k}(\varphi)$ is said to be an essential set of $F_{k}(\varphi)$ with respect to $\rho_{1}$ (or $\rho(m)$) if, given any number $\epsilon>0$, there exists a corresponding number $\delta >0$ such that $F_{k}(\varphi^{\prime})\cap[e(\varphi)+B_{\epsilon }(0)]\neq\emptyset$ for all $\varphi^{\prime}\in\mathcal{M}$ such that $\rho_{1}(\varphi^{\prime},\varphi)<\delta$ (or $\rho_{m}(\varphi ^{\prime},\varphi)< \delta$).

### Remark 2

Similar to the above Remark 1, we can define the essential point and essential component with respect to $\rho_{1}$ (or $\rho(m)$).

The following example (Example 4.1, [26]) illustrates that the essence of the set of Ky Fan’s points with respect to the metric $\rho_{1}$ is not necessarily associated with the essence of it with respect to the uniform metric $\rho_{m}$.

### Example 1

Let $X=[0,1]$, $\varphi:X\times X\rightarrow R$ and $\varphi(x,y)=0$ for all $(x,y)\in X\times X$. Then, $\varphi\in\mathcal{M}$ and $E_{\varphi}(x)=X=[0,1]$.

**Figure 1:**
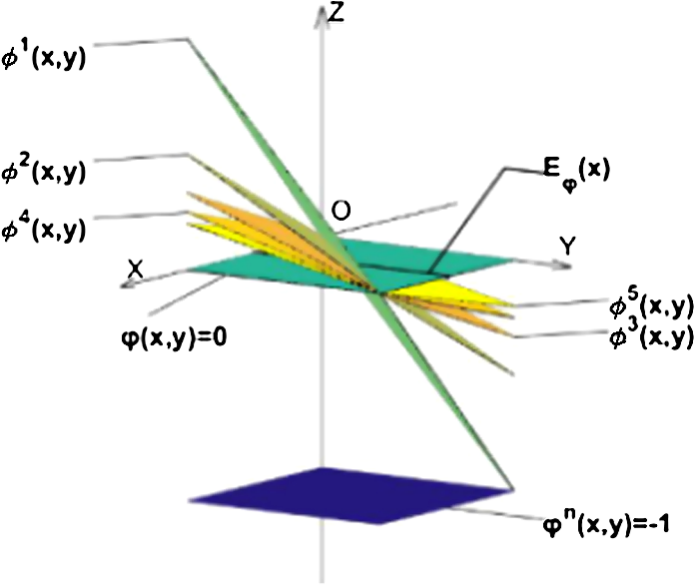
**The graph of funtion** ***φ*** **,** $\pmb{\varphi^{n}}$ **,** $\pmb{\phi^{1}}$ **,** $\pmb{\phi^{2}}$ **,** $\pmb{\phi^{3}}$ **,** $\pmb{\phi^{4}}$ **and** $\pmb{\phi^{5}}$ **.**

On the one hand,for $n=1,2,\ldots $ , define $\varphi^{n}:X\times X\rightarrow R$ and $\varphi^{n}(x,y)=-1$ for all $(x,y)\in X\times X$. Then, $\varphi^{n} \in\mathcal{M}$ and $E_{\varphi ^{n}}(x)=X=[0,1]$ (see Figure 1).It is clear that $\rho_{1}(\varphi^{n},\varphi)\rightarrow0$ while $\rho_{m}(\varphi^{n},\varphi)=1$ does not converge to 0.

On the other hand,for $n=1,2,\ldots $ , define $\phi^{n}:X\times X\rightarrow R$ and $\phi ^{n}(x,y)=\frac{1}{n} x-\frac{1}{n} y$ for all $(x,y)\in X\times X$. Then, $\phi^{n}\in\mathcal{M}$ and $E_{\phi^{n}}(x)=[x,1]$ (see Figure 1). it is clear that $\rho_{m}(\phi^{n},\varphi)\rightarrow0$ while $\rho_{1}(\phi^{n},\varphi)=1$ does not converge to 0.

Therefore, the essence of the set of Ky Fan’s points is not necessarily associated with these two types of perturbation defined by the metric $\rho_{1}$ and $\rho_{m}$, respectively. This illustrates that the perturbation of the inequality functions, even defined by the strong sup-norm metric, when it is sufficiently small, cannot guarantee that the perturbation of their section mappings is sufficiently small.

How can a type of perturbation be defined such that it includes these two types of perturbation? To answer this question, we propose a weaker metric to establish a stronger type of perturbation.

For the section mappings and the inequality functions, we also define the symmetric semi-metrics by the formula

$$\rho_{s}^{u}(E_{A},E_{B})=\sup _{x\in X}H_{u}\bigl(E_{A}(x),E_{B}(x) \bigr) \quad(E_{A},E_{B}\in\mathcal{E}) $$

and

$$\rho_{k}^{u}(\varphi_{1},\varphi_{2})= \rho_{s}^{u}(E_{\varphi _{1}},E_{\varphi_{2}})=\sup _{x\in X}H_{u}\bigl(E_{\varphi_{1}}(x),E_{\varphi _{2}}(x) \bigr) \quad(\varphi_{1},\varphi_{2}\in\mathcal{M}), $$

where $H_{u}(X_{2},X_{1})=\sup_{x\in X_{2}}d(x,X_{1})$ is the Hausdorff upper semi-metric.

Because $H_{u}(X_{2},X_{1})=\max\{ H_{u}(X_{2},X_{1}),H_{l}(X_{2},X_{1})\}$, we have the following results.

### Proposition 1

*Let* $\varphi\in\mathcal{M}$ *and* $\{\varphi ^{n}\} \subset\mathcal{M}$,

1. $\rho_{s}^{u}\le\rho_{s}$, $\rho_{k}^{u}\le\rho_{1}$.
2. *If* $\rho_{m}(\varphi^{n},\varphi)\rightarrow0$ ($n\rightarrow\infty $), *then* $\rho_{k}^{u}(\varphi^{n},\varphi)\rightarrow0$.

### Proof

(1) It follows immediate from the definition of $\rho_{1}$, $\rho_{s}$, $\rho_{s}^{u}$ and $\rho_{k}^{u}$.

(2) In fact, if (2) does not hold, *i.e.*, to given a number $\epsilon_{0}>0$, there exists a sequence of positive numbers $\{\delta _{n}\}$ convergent to 0 such that $\rho_{k}^{u}(\varphi^{n},\varphi)\ge \epsilon_{0}$ for all $\varphi^{n},\varphi\in\mathcal{M}$ such that $\rho_{m}(\varphi^{n},\varphi)<\delta_{n}$. By using that $H_{u}(E_{\varphi^{n}},E_{\varphi})=\sup_{x\in E_{\varphi ^{n}}}d(x,E_{\varphi})\ge\epsilon_{0}$, we see that there exist $x_{0}\in X$ and $y_{n}\in X$ such that $y_{n}\in E_{\varphi ^{n}}(x_{0})$ and $d(y_{n},E_{\varphi}(x_{0}))\ge\epsilon_{0}$. Note that $E_{\varphi}(x)= \{y\in X|\varphi(x,y)\leq0\}$ and $E_{\varphi ^{n}}(x)=\{y\in X|\varphi^{n}(x,y)\leq0\}$, we see that $\varphi ^{n}(x_{0},y_{n})\leq0$. Since *X* is compact, without loss of generality, we may assume that $y_{n}\rightarrow y_{0}$. Because $\rho_{m}(\varphi _{n},\varphi)\rightarrow0$, we see that $\varphi(x_{0},y_{0})\le0$ and $y_{0}\in E_{\varphi}(x_{0})$. Thus, for enough large *n*, we see that $y_{n}\in E_{\varphi}(x_{0})$. Hence, $d(y_{n},E_{\varphi}(x_{0}))\ge \epsilon_{0}$ does not hold, which is a contradiction. Therefore, (2) holds. □

Proposition 1 illustrates that the perturbation defined by $\rho _{k}^{u}$ includes two perturbations defined by $\rho_{1}$ and $\rho _{m}$, *i.e.*, the perturbation with respect to $\rho_{k}^{u}$ is sufficiently small whenever the perturbation with respect to $\rho _{1}$ and $\rho_{m}$ is sufficiently small. In Example 1, it is clear that $E_{\varphi^{n}}(x)\subset E_{\varphi}(x)$ and $E_{\phi ^{n}}(x)\subset E_{\varphi}(x)$. Hence, $\rho_{k}^{u}(\varphi ^{n},\varphi)\rightarrow0$ and $\rho_{k}^{u}(\phi^{n},\phi)\rightarrow0$.

## Stability results on Ky Fan’s theorems

In this section, we first introduce the concepts of the essential solution of Ky Fan’s section theorem and the essential Ky Fan point with respect to $\rho_{s}^{u}$ and $\rho_{k}^{u}$.

### Definition 3

Let $E\in\mathcal{E} $. A nonempty closed subset $e(E)$ of $F_{s}(E)$ is said to be an essential set of $F_{s}(E)$ with respect to $\rho_{s}^{u}$ if, given any number $\epsilon>0$, there exists a corresponding number $\delta>0 $ such that $F_{s}(E^{\prime })\cap[e(E)+B_{\epsilon} (0)]\neq\emptyset$ for all $E^{\prime}\in\mathcal{E} $ such that $\rho_{s}^{u}(E^{\prime },E)<\delta$.

### Definition 4

Let $\varphi\in\mathcal{M}$. A nonempty closed subset $e(\varphi)$ of $F_{k}(\varphi)$ is said to be an essential set of $F_{k}(\varphi)$ with respect to $\rho_{k}^{u}$ (or $\rho_{m}$) if, given any number $\epsilon>0$, there exists a corresponding number $\delta>0 $ such that $F_{k}(\varphi^{\prime})\cap[e(\varphi )+B_{\epsilon} (0)]\neq\emptyset$ for all $\varphi^{\prime}\in\mathcal{M}$ such that $\rho _{k}^{u}(\varphi^{\prime},\varphi)<\delta$.

### Remark 3

(1) By Definition 3, Definition 4 and Proposition 1, an essential set with respect to $\rho_{s}^{u}$ is clearly an essential set with respect to $\rho_{s}$, an essential set with respect to $\rho _{k}^{u}$ is clearly an essential set with respect to $\rho_{m}$ (or $\rho_{1}$).

(2) If $\mathcal{S}$ is a minimum element of the family of all the essential sets with a partial order defined by the inclusion relation, then $\mathcal{S}$ is said to be a minimum essential set. $\mathcal{S}$ is said to be a stable set, if $\mathcal{S}$ is a minimum essential set and connected.

Let $F:Z\rightarrow2^{Y}$ be a set-valued mapping with nonempty values. *F* is said to be upper semicontinuous at $z\in Z$ if, given any number $\epsilon>0$, there exists a corresponding number $\delta>0 $ such that $F(z^{\prime})\subset[F(z)+B_{\epsilon}(0)]$ for all $z^{\prime}\in Z $ such that $d(z^{\prime},z)<\delta$. *F* is said to be a usco mapping, if *F* is upper semicontinuous on *Z* and compact-valued.

### Lemma 1

1. $F_{s}:(\mathcal{E} ,\rho_{s}^{u})\rightarrow K(X)$ *is a usco mapping*.
2. $F_{k}:(\mathcal{M},\rho_{k}^{u})\rightarrow K(X)$ *is a usco mapping*.

### Proof

(1) In fact, if (1) does not hold, *i.e.*, to given a number $\epsilon_{0}>0$, there exists a sequence of positive numbers $\{ \delta_{n}\}$ convergent to 0 such that $y_{n}\in F_{s}(E^{n})$ and $y_{n}\notin[F_{s}(E)+B_{\epsilon_{0}}(0)]$ for all $E^{n}\in\mathcal {E} $ such that $\rho_{s}^{u}(E^{n},E)<\delta_{n}$. Since *X* is compact, without loss of generality, we may assume that $y_{n}\rightarrow y$. Hence, $y_{n}\notin[F_{s}(E)+B_{\epsilon _{0}}(0)]$ implies $y\notin[F_{s}(E)+B_{\epsilon_{0}}(0)]$. Thus, we see that $y\notin F_{s}(E)$. For every $x\in X$, since $y_{n}\in F_{s}(E^{n})$, we see that $(x,y_{n}) \in A^{n} $, which implies that $y_{n}\in E^{n}(x)$. By using that $\rho_{s}^{u}(E^{n},E)=\sup_{y\in E^{n}}d(y,E)<\delta_{n}$, we see that there exists a sequence $\{ y_{n}^{1}\}$ of $E(x)$ such that $d(y_{n},y_{n}^{1})<\delta_{n}$. Note that $y_{n}\rightarrow y$, we see that $y_{n}^{1}\rightarrow y$. By the closeness of $E(x)$, we see that $y\in E(x)$, which implies that $y\in \bigcap_{x\in X}E(x)$, by using that $\bigcap_{x\in X}E(x)=F_{s}(E)$, we see that $y\in F_{s}(E)$. Hence, $y\notin F_{s}(E)$ does not hold, which is a contradiction. Therefore, (1) holds.

(2) From (1), to given any number $\epsilon>0$, there exists a corresponding number $\delta>0$ such that $F_{s}(E^{\prime})\subset [F_{s}(E)+B_{\epsilon}(0)]$ for all $E^{\prime}\in\mathcal{E} $ such that $\rho_{s}^{u}(E^{\prime},E)<\delta$. By Definition of $\rho _{k}^{u}$, if $\rho_{k}^{u}(\varphi^{\prime},\varphi)<\delta$, then $\rho_{k}^{u}(\varphi^{\prime},\varphi)=\rho_{k}^{u}(E_{\varphi^{\prime }},E_{\varphi})<\delta$ and $F_{s}(E_{\varphi^{\prime}})\subset [F_{s}(E_{\varphi})+B_{\epsilon}(0)]$. Note that $F_{k}(\varphi )=F_{s}(E_{\varphi})$ and $F_{k}(\varphi^{\prime})=F_{s}(E_{\varphi ^{\prime}})$, we see that $F_{k}(\varphi^{\prime})\subset[F_{k}(\varphi )+B_{\epsilon}(0)]$. Hence, $F_{k}$ is a usco mapping. □

### Lemma 2

*For every* $E \in\mathcal{E} $, $F_{s}(E)$ *has at least one minimum essential set with respect to* $\rho_{s}^{u}$.

### Proof

For every $E\in\mathcal{E} $, by Lemma 1,we see that there exists a number $\delta>0$ such that $F_{s}(E^{\prime})\subset[F_{s}(E)+B_{\epsilon}(0)]$ for all $E^{\prime}\in\mathcal{E} $ such that $\rho_{s}^{u}(E^{\prime},E)<\delta$. Thus, we see that $F_{s}(E^{\prime})\cap[F_{s}(E)+B_{\epsilon}(0)]\neq\emptyset$. Hence, $F_{s}(E)$ is the essential set with respect to $\rho_{s}^{u}$. □

Let $\mathcal{S}$ denote the set of all essential sets of $F_{s}(E)$, then $\mathcal{S}\neq\emptyset$. If the sets in $\mathcal{S}$ are ordered by set inclusion, then $\mathcal{S}$ is a partially set. For any decreasing chain $\mathcal{C}$ in $\mathcal{S}$, since all the sets in $\mathcal{C}$ are compact, $\bigcap_{c\in\mathcal{C}} C$, denoted by *D*, is nonempty, and *D* is a lower bound of $\mathcal{C}$. By Zorn’s lemma, there must be a minimum element *s* in $\mathcal{S}$, and *s* is just a minimum essential set of $F_{s}(E)$.

### Theorem 1

*For every* $E\in\mathcal{E} $, *if the minimum essential set of* $F_{s}(E)$ *with respect to* $\rho_{s}^{u}$ *is connected*, *then it is a stable set*.

### Proof

For every $E\in\mathcal{E} $, by Lemma 2, let $m(E)$ be a minimum essential subset of $F_{s}(E)$ with respect to $\rho_{s}^{u}$. If $m(E)$ is not connected, then there exist two nonempty compact subsets $C_{1}(E)$, $C_{2}(E)$ and two disjoint open subsets $V_{1}$, $V_{2}$ of *X* such that $m(E)=C_{1}(E)\cup C_{2}(E)$ and $V_{1}\supset[C_{1}(E)+B_{\epsilon }(0)]$, $V_{2}\supset[C_{2}(E)+B_{\epsilon}(0)]$ for any number $\epsilon>0$. Since $m(E)$ is a minimum essential set of $F_{s}(E)$, neither $C_{1}(E)$ nor $C_{2}(E)$ is essential with respect to $\rho _{s}^{u}$. There exists a number $\epsilon_{0}>0$, such that, to any sequence of positive numbers $\{\delta_{n}\}$ convergent to 0, there exist $E_{n}^{1},E_{n}^{2}\in\mathcal{E} $ such that

$$\rho_{s}^{u}\bigl(E_{n}^{1},E\bigr)< \delta_{n}, \qquad\rho_{s}^{u}\bigl(E_{n}^{2},E \bigr)< \delta _{n},\quad\quad F_{s}\bigl(E_{n}^{1} \bigr)\cap V_{1}=\emptyset,\qquad F_{s} \bigl(E_{n}^{2}\bigr)\cap V_{2}= \emptyset. $$

Next, define $E_{n}^{\prime}:X\rightarrow K(X)$ as follows:

$$E_{n}^{\prime}(x)=\bigl(E_{n}^{1}(x)\setminus V_{2}\bigr)\cup \bigl(E_{n}^{2}(x) \setminus V_{1}\bigr) \quad(x\in X). $$

We now check that $E_{n}^{\prime} \in\mathcal{E} $. Let $A_{n}^{\prime }=\{(x,y)\in X\times X|y\in E_{n}^{\prime}(x) \}$, we check only that $A_{n}^{\prime}$ satisfies conditions (1)-(3) from Theorem A.

(i) Since $A_{n}^{1}$, $A_{n}^{2}$ satisfy condition (1) from Theorem A and $V_{1}$, $V_{2}$ are two open sets, we know that $\{y\in X|(x,y)\in A_{n}^{\prime} \}=E_{n}^{\prime }(x)=(E_{n}^{1}(x)\setminus V_{2})\cup(E_{n}^{2}(x)\setminus V_{1})$ is a closed set for every $x\in X$.
(ii) For every $y\in X$, since
  $$\begin{aligned} \bigl\{ x\in X|(x,y)\notin A_{n}^{\prime} \bigr\} &=\bigl\{ x\in X|y \notin E_{n}^{\prime }(x) \bigr\} \\&=\bigl\{ x\in X|y\notin\bigl[ \bigl(E_{n}^{1}(x)\setminus V_{2}\bigr)\cup \bigl(E_{n}^{2}(x)\setminus V_{1}\bigr) \bigr]\bigr\} ,\end{aligned} $$
  note that $V_{1}\cap V_{2}\subset\overline{V_{1}} \cap \overline{V_{1}}=\emptyset$, if $y\in V_{1}$, then
  $$\begin{aligned} \bigl\{ x\in X|(x,y)\notin A_{n}^{\prime} \bigr\} &=\bigl\{ x\in X|y \notin \bigl[\bigl(E_{n}^{1}(x)\setminus V_{2} \bigr)\cup \bigl(E_{n}^{2}(x)\setminus V_{1} \bigr)\bigr]\bigr\} \\&=\bigl\{ x\in X|y\notin E_{n}^{1}(x) \bigr\} = \bigl\{ x\in X|(x,y)\notin A_{n}^{1} \bigr\} ;\end{aligned} $$
  and if $y\in V_{2}$, then
  $$\begin{aligned} \bigl\{ x\in X|(x,y)\notin A_{n}^{\prime} \bigr\} &=\bigl\{ x\in X|y \notin \bigl[\bigl(E_{n}^{1}(x)\setminus V_{2} \bigr)\cup\bigl(E_{n}^{2}(x)\setminus V_{1}\bigr)\bigr]\bigr\} \\&=\bigl\{ x\in X|y\notin E_{n}^{2}(x) \bigr\} =\bigl\{ x\in X|(x,y)\notin A_{n}^{2} \bigr\} ,\end{aligned} $$
  hence, in either case, $\{x\in X|(x,y)\notin A_{n}^{\prime} \}$ is convex set or empty for every $y\in X$.
(iii) For every $x\in X$, if $(x,x)\in A_{n}^{\prime} $ is not true, then there exists $(x_{0},x_{0})\notin A_{n}^{\prime} $ such that $x_{0}\notin E_{n}^{\prime} (x_{0})$, *i.e.*, $x_{0}\notin [(E_{n}^{1}(x_{0})\setminus V_{2})\cup(E_{n}^{2}(x_{0})\setminus V_{1})]$. Since $x_{0}\in E_{n}^{1}(x_{0})$ and $x_{0}\in E_{n}^{2}(x_{0})$, we deduce that $x_{0}\in V_{1}\cap V_{2}$. Hence, $V_{1}\cap V_{2}=\emptyset$ does not hold, which is a contradiction. Moreover, $A_{n}^{\prime} $ satisfies conditions (1)-(3) from Theorem A, then $E_{n}^{\prime}\in\mathcal{E} $.

To prove by contraposition that $F(E_{n}^{\prime})\cap(V_{1}\cup V_{2})=\emptyset$. Suppose the existence of $y_{0}\in X$ such that $y_{0}\in[F_{s}(E_{n}^{\prime})\cap(V_{1}\cup V_{1})]$, which implies that $y_{0}\in V_{1}\cup V_{2}$. Without loss of generality, we may assume that $y_{0}\in V_{1}$. Since $F_{s}(E_{n}^{1})\cap V_{1}=\emptyset$, we see that $y_{0}\notin F_{s}(E_{n}^{1})$ and there exists $x_{0}\in X$ such that $y_{0}\notin E_{n}^{1}(x_{0})$. Note that $y_{0}\in F_{s}(E_{n}^{\prime})$, we see that $y_{0}\in E_{n}^{\prime}(x_{0})$, which implies that $y_{0}\in E_{n}^{2}(x_{0})\setminus V_{1}$. Thus, we see that $y_{0}\notin V_{1}$. Hence, $y_{0}\in V_{1}$ does not hold, which is a contradiction.

Finally, we check that $E_{n}^{\prime}(x)\subset[E(x)+B_{\delta _{n}}(0)]$ for every $x\in X$. In fact,

$$E_{n}^{\prime}(x)=\bigl(E_{n}^{1}(x)\setminus V_{2}\bigr)\cup \bigl(E_{n}^{2}(x)\setminus V_{1}\bigr)\subset E_{n}^{1}(x)\cup E_{n}^{2}(x), $$

and $\rho_{s}^{u}(E_{n}^{1},E)<\delta_{n}$, $\rho _{s}^{u}(E_{n}^{2},E)<\delta_{n}$, thus $E_{n}^{1}(x)\subset[E(x)+B_{\delta_{n}}(0)]$, $E_{n}^{2}(x)\subset [E(x)+B_{\delta_{n}}(0)]$.

Consequently,

$$E_{n}^{\prime}(x)\subset E_{n}^{1}(x)\cup E_{n}^{2}(x)\subset \bigl[E(x)+B_{\delta_{n}}(0)\bigr], $$

that is, $\rho_{s}^{u}(E_{n}^{\prime},E)<\delta_{n}$. It follows that $F_{s}(E_{n}^{\prime})\cap[m(E)+B_{\epsilon}(0)]=F_{s}(E_{n}^{\prime })\cap[(C_{1}(E)\cup C_{2}(E))+B_{\epsilon}(0)]\subset F_{s}(E_{n}^{\prime })\cap(V_{1}\cup V_{2})=\emptyset$, which contradicts the fact that $m(E)$ is essential with respect to $\rho_{s}^{u}$. Hence, the minimum essential set $m(E)$ is connected. □

### Theorem 2

*For every* $E\in\mathcal{E} $, $F_{s}(E)$ *has at least one essential connected component with respect to* $\rho_{s}^{u}$.

### Proof

For every $E\in\mathcal{E} $, by Lemma 2 and Theorem 1, we see that there exists at least one connected minimum essential set $m(E)$ of $F_{s}(E)$. By the connectivity of $m(E)$, we see that $m(E)$ must be included in a component $C_{\alpha}$ of $F_{s}(E)$. Since $m(E)\subset C_{\alpha}$, we see that $C_{\alpha}$ is an essential sets of $F_{s}(E)$ with respect to $\rho_{s}^{u}$. Thus, $C_{\alpha}$ is an essential component of $F_{s}(E)$. □

By Theorems 1 and 2, we can prove the corresponding results on Ky Fan’s point and especially the existence of the essential component.

### Theorem 3

*For every* $\varphi\in\mathcal{M}$, $F_{k}(\varphi )$ *has at least one essential component with respect to* $\rho_{k}^{u}$.

### Proof

For every $\varphi\in\mathcal{M}$, let $E_{\varphi}\in \mathcal{E} $ be a corresponding section mapping. By Theorem 2, we see that $F_{s}(E_{\varphi})$ has one essential component with respect to $\rho_{s}^{u}$, denoted by $C_{\alpha}$. Since $C_{\alpha}$ is essential, to given any number $\epsilon>0$, there exists a corresponding number $\delta>0$ such that $F_{s}(E^{\prime})\cap [C_{\alpha}+ B_{\epsilon}(0)]\neq\emptyset$ for all $E^{\prime}\in\mathcal{E}$ such that $\rho_{s}^{u}(E^{\prime },E_{\varphi})<\delta$. Thus, for all $\varphi^{\prime}\in\mathcal {M}$, if $\rho_{k}^{u}(\varphi^{\prime},\varphi)<\delta$, then $\rho _{s}^{u}(E_{\varphi^{\prime}},E_{\varphi})=\rho_{k}^{u}(\varphi^{\prime },\varphi)<\delta$, thus $F_{s}(E_{\varphi})\cap[m(E_{\varphi })+B_{\epsilon}(0)]\neq\emptyset$. Note that $F_{k}(\varphi )=F_{s}(E_{\varphi})$, we see that $F_{s}(E_{\varphi})\cap [m(E_{\varphi})+B_{\epsilon}(0)]\neq\emptyset$. By Definition 2, we see that $C_{\alpha}$ is an essential component of $F_{k}(\varphi)$ with respect to $\rho_{k}^{u}$. □

By Proposition 1 and Theorems 2 and 3, we may deduce the existence of the essential components of the solutions of Ky Fan’s section theorem with respect to $\rho_{s}$ and the essential components of Ky Fan’s points with respect to $\rho_{m}$ or $\rho_{1}$.

### Corollary 1

*For every* $E\in\mathcal{E} $, $F_{s}(E)$ *has at least one essential component with respect to* $\rho_{s}$.

### Proof

For every $E\in\mathcal{E} $, by Theorem 2, we see that $F_{s}(E)$ has at least one essential component with respect to $\rho _{s}^{u}$, denoted by $C_{\alpha}$. Since $C_{\alpha}$ is essential, to given any number $\epsilon>0$, there exists a corresponding number $\delta>0$ such that $F_{s}(E^{\prime})\cap[C_{\alpha}+B_{\epsilon }(0)]\neq\emptyset$ for all $E^{\prime}\in\mathcal{E} $ such that $\rho_{s}^{u}( E^{\prime},E)<\delta$. By result (1) from Proposition 1, there exists a number $\eta>0$ such that $\rho_{s}^{u}(E^{\prime },E)<\delta$ for all $E^{\prime}\in\mathcal{E} $ such that $\rho _{u}(E^{\prime},E)<\eta$. Thus, we see that $F_{s}(E^{\prime})\cap [C_{\alpha}+B_{\epsilon}(0)]\neq\emptyset$. Moreover, by Definition 2, we see that $C_{\alpha}$ is the essential components of $F_{s}(E)$ with respect to $\rho_{s}$. □

### Corollary 2

*For every* $\varphi\in\mathcal{M}$, $F_{k}(\varphi )$ *has at least one essential connected component with respect to* $\rho_{m}$.

### Proof

The proof of Corollary 2 is similar to Corollary 1. □

### Corollary 3

*For every* $\varphi\in\mathcal{M}$, $F_{k}(\varphi )$ *has at least one essential connected component with respect to* $\rho_{1}$.

### Proof

The proof of Corollary 3 is similar to Corollary 1. □

## Stability results on the Nash equilibrium

Suppose that we have a finite set *N* of *n* plays. Let $X_{i}$ be the finite set of pure strategies of player $i\in N$. We define $X=\prod_{i=1}^{n} X_{i}$. For all $i\in N$, let $f_{i}:X\rightarrow R$ be the payoff function of player *i*. We define a 2*N*-tuple $(X_{1},\ldots,X_{n};f_{1},\ldots,f_{n})$ as an *n*-person noncooperative game, denoted by $\Gamma(X,f)$. For arbitrary $x=(x_{1},\ldots,x_{n})\in X$, we define $x_{-i}=(x_{1},\ldots,x_{i-1},x_{i+1},\ldots,x_{n})$.

An *N*-tuple $x^{\ast}\in X$ is a Nash equilibrium point of $\Gamma (X,f)$ if for all $i\in N$ we see that

$$f_{i}\bigl(x_{i}^{\ast},x_{-i}^{\ast} \bigr)=\max_{y_{i}\in X_{i}}f_{i}\bigl(y_{i},x_{-i}^{\ast} \bigr)\quad(y_{i}\in X_{i}). $$

We introduce now the collective-best-reply correspondence of $\Gamma (X,f)$ ([12]).

Define $U:X\times X\rightarrow R$ as follows:

$$U(x,y)=\sum_{i\in N} f_{i}(x_{i},y_{-i}) \quad(x,y\in X), $$

then the collective-best-reply correspondence $\mathit{CBR}_{\Gamma (X,f)}:X\rightarrow X$ is defined as

$$\mathit{CBR}_{\Gamma(X,f)}(x)=\bigl\{ y\in X|U(x,y)\leq U(y,y)\bigr\} \quad(x\in X). $$

Let

$$\psi_{f}(x,y)=U(x,y)-U(y,y)=\sum_{i\in N} f_{i}(x_{i},y_{-i})-\sum _{i\in N} f_{i}(y_{i},y_{-i}) \quad(x,y\in X), $$

then

$$\mathit{CBR}_{\Gamma(X,f)}(x)=\bigl\{ y\in X|\psi_{f}(x,y)\leq0\bigr\} \quad(x \in X). $$

Let $\Gamma(X,f)$ be a game with the following properties:

1. For all $i\in N$, $X_{i}$ is a nonempty compact convex subset in a normed linear space $E_{i}$.
2. For all $i\in N$, $\sum_{i\in N} f_{i}$ is upper semicontinuous on *X*.
3. For all $i\in N$ and any fixed $x_{-i}\in X_{-i}$, $f_{i}(x_{i},\cdot)$ is lower semicontinuous on $X_{-i}$.
4. For all $i\in N$ and any fixed $y_{-i}\in X_{-i}$, $\sum_{i\in N}f_{i}(\cdot,y_{-i})$ is quasi-concave on *X*.

We discuss now the stability of the Nash equilibrium for the fixed *X*. Denote $\Gamma(X,f)$ by $\Gamma(f)$, and we denote by $\mathcal {G}$ the collection of all $\Gamma(f)$ such that all conditions of $\Gamma(X,f)$ hold.

For every $\Gamma(f)\in \mathcal{G}$, it is easy to verify that $\psi _{f}\in \mathcal{M}$ and $\mathit{CBR}_{\Gamma(f)}(x)=E_{\psi_{f}}$, that is, $\psi_{f}(x,y)$ satisfies all conditions of Ky Fan’s inequality theorem. Additionally, using Definition of the Nash equilibrium, we see that $x^{*}\in F_{k}(\psi_{f})$ iff $x^{*}$ is a Nash equilibrium point of $\Gamma(f)$. Denote the set of all Nash equilibrium points of $\Gamma(f)$ by $N(\Gamma(f))$, then $N(\Gamma (f))=F_{k}(\psi_{f})$. Note that $F_{k}(\psi_{f})\neq\emptyset$, we see that $N:\mathcal{G}\rightarrow K(X)$.

In $\mathcal{G}$, define the sup-norm metric of the payoff function and the maximum Hausdorff metric of the collective-best-reply correspondence ([23–26]) by

$$\rho_{m}\bigl(\Gamma(f),\Gamma(g)\bigr)=\sup_{x\in X}\big|f(x)-g(x)\big| $$

and

$$\begin{aligned} \rho_{u}\bigl(\Gamma(f),\Gamma(g)\bigr)&=\sup_{x\in X}H_{d} \bigl(\mathit{CBR}_{\Gamma (f)}(x),\mathit{CBR}_{\Gamma(g)}(x)\bigr)\\& =\sup_{x\in X}H_{d} \bigl(E_{\psi_{f}}(x),E_{\psi_{g}}(x)\bigr)\quad\bigl(\Gamma (f),\Gamma(g) \in\mathcal{G}\bigr).\end{aligned} $$

Using Definition of the metric, we see that $\rho_{\Gamma}(\Gamma (f),\Gamma(g))=\rho_{u}^{k}(\psi_{f},\psi_{g})$.

Further, define the maximum Hausdorff semi-metric by

$$\rho_{\Gamma}^{u}\bigl(\Gamma(g),\Gamma(f)\bigr)= \rho_{s}^{u}\bigl(E_{\psi _{g}}(x),E_{\psi_{f}}(x) \bigr)=\rho_{k}^{u}(\psi_{g},\psi_{f}). $$

### Definition 5

Let $\Gamma(f)\in\mathcal{G}$. A nonempty closed set $N(\Gamma(f))$ is an essential set with respect to $\rho _{\Gamma}^{u}$ if, given any number $\epsilon>0$, there exists a corresponding number $\delta>0 $ such that $N(\Gamma(g))\cap [N(\Gamma(f))+B_{\epsilon}(0)]\neq\emptyset$ for all $\Gamma(g)\in\mathcal{G}$ such that $\rho_{\Gamma}^{u}(\Gamma (g),\Gamma(f))<\delta$.

By Theorem 3, we obtain immediately the existence of the essential components of the Nash equilibrium with respect to $\rho_{\Gamma}^{u}$.

### Theorem 4

*For every* $\Gamma(f)\in\mathcal{G}$, $N(\Gamma (f))$ *has at least one essential component with respect to* $\rho_{\Gamma}^{u}$.

### Proof

For every $\Gamma(f)\in\mathcal{G}$, by Theorem 3, we see that $F_{k}(\psi_{f})$ has at least one essential component with respect to $\rho_{\Gamma}^{u}$, denoted by $C_{\alpha}$. Since $C_{\alpha}$ is essential, to given any number $\epsilon>0$, there exists a corresponding number $\delta>0$ such that $F_{k}(\psi^{\prime })\cap[C_{\alpha}+B_{\epsilon}(0)]\neq\emptyset$ for all $\psi ^{\prime}\in\mathcal{M}$ such that $\rho_{k}^{u}(\psi^{\prime},\psi _{f})<\delta$. If $\rho_{\Gamma}^{u}(\Gamma(g), \Gamma(f))<\delta$, then $\rho_{k}^{u}(\psi_{g},\psi_{f})<\delta$ and $F_{k}(\psi _{g})\cap[C_{\alpha}+B_{\epsilon}(0)]\neq\emptyset$. By using that $F_{k}(\psi_{g})=N(\Gamma(g))$, we see that $N(\Gamma(g))\cap [C_{\alpha}+B_{\epsilon}(0)]\neq\emptyset$. Moreover, we see that $C_{\alpha}$ is an essential component of $N(\Gamma(f))$ with respect to $\rho_{\Gamma}^{u}$. □

The perturbation of $\Gamma(f)$ is defined by the inequality functions corresponding to the payoff functions of $\Gamma(f)$, therefore, by using result (1) from Proposition 1, we see that the essential component with respect to $\rho_{\Gamma}^{u}$ is an essential component with respect to $\rho_{m}(\Gamma(f),\Gamma(g))$ and $\rho_{u}(\Gamma(f),\Gamma (g))$, and it has stronger stability.

## Conclusions

Based on the stronger type of perturbation of the section mappings defined by a semi-metric called the maximum Hausdorff semi-metric, some further results on the stability of Ky Fan’s points are proposed in Section 3, which include the existence of the essential components of the set of Ky Fan’s points to this perturbation. By Example 1 and Proposition 1, the essential component with respect to the maximum Hausdorff semi-metric is a unified frame to describe the stability based on the sup-norm metric and maximum Hausdorff metric, and it has stronger stability. In Section 4,we also give an example to illustrate our results.
